# scDown: A Pipeline for Single-Cell RNA-Seq Downstream Analysis

**DOI:** 10.3390/ijms26115297

**Published:** 2025-05-30

**Authors:** Liang Sun, Qianyi Ma, Chunhui Cai, Maryam Labaf, Ashish Jain, Caroline Dias, Shira Rockowitz, Piotr Sliz

**Affiliations:** 1 Research Informatics, Department of Information Technology, Boston Children’s Hospital, Boston, MA 02115, USA; liang.sun@childrens.harvard.edu (L.S.);; 2 Department of Pediatrics, Section of Developmental Pediatrics, Section of Genetics and Metabolism, Children’s Hospital Colorado, University of Colorado Anschutz Medical Campus, Aurora, CO 80045, USA; 3 Children’s Rare Disease Collaborative, Boston Children’s Hospital, Boston, MA 02115, USA; 4 Division of Genetics and Genomics, Boston Children’s Hospital, Boston, MA 02115, USA; 5 Department of Pediatrics, Harvard Medical School, Boston, MA 02115, USA; 6 The Manton Center for Orphan Disease Research, Boston Children’s Hospital, Boston, MA 02115, USA; 7 Division of Molecular Medicine, Boston Children’s Hospital, Boston, MA 02115, USA; 8 Department of Biological Chemistry and Molecular Pharmacology, Harvard Medical School, Boston, MA 02115, USA

**Keywords:** single-cell transcriptomics, cell–cell communication, pseudotime analysis, trajectory analysis, cell proportion difference analysis

## Abstract

Single-cell transcriptomics data are analyzed using two popular tools, Seurat and Scanpy. Multiple separate tools are used downstream of Seurat and Scanpy cell annotation to study cell differentiation and communication, including cell proportion difference analysis between conditions, pseudotime and trajectory analyses to study cell transition, and cell–cell communication analysis. To automate the integrative cell differentiation and communication analyses of single-cell RNA-seq data, we developed a single-cell RNA-seq downstream analysis pipeline called “scDown”. This R package includes cell proportion difference analysis, cell–cell communication analysis, pseudotime analysis, and RNA velocity analysis. Both Seurat and Scanpy annotated single-cell RNA-seq data are accepted in this pipeline. We applied scDown to a published dataset and identified a unique, previously undiscovered signature of neuronal inflammatory signaling associated with a rare genetic neurodevelopmental disorder. These findings were not identified with a simple implementation of Seurat differential gene expression analysis, illustrating the value of our pipeline in biological discovery. scDown can be broadly utilized in downstream analyses of scRNA-seq data, particularly in rare diseases.

## 1. Introduction

Over 10,000 rare diseases affect more than 30 million Americans, according to the National Organization for Rare Disorders (NORD) database. These diseases often lack effective treatments due to their complex and heterogeneous cell populations. For example, rare diseases such as Amyotrophic Lateral Sclerosis, Interstitial Lung Disease, and Glioblastoma involve a complex interplay of various cell types within their cellular environments [1,2,3]. With advances in single-cell RNA sequencing (scRNA-seq) technology and the reduced costs of sequencing, scRNA-seq has become one of the most widely used methods for analyzing gene expression at single-cell resolution. This technology is increasingly applied to understand disease etiologies including rare genetic disorders. Standard pipelines for scRNA-seq analysis involve preprocessing steps such as quality control, read alignment to a reference genome, normalization, dimensionality reduction, cell clustering, cell type annotation, and differential expression analysis between conditions. However, meaningful biological insights often require further downstream analysis including cell proportion difference analysis to determine whether specific cell types are differentially abundant between conditions; pseudotime analysis to model cellular differentiation and development; RNA velocity analysis to predict future cellular states based on spliced and unspliced transcript ratios; and cell–cell communication analysis to infer intercellular signaling networks. Most projects necessitate multiple analyses to fully resolve the biological complexity.

Although multiple tools exist for each of these analyses, performing them requires users to install, learn, and integrate multiple software packages. To address this limitation, we developed scDown, an R package version 1 (https://github.com/BCH-RC/scDown, accessed on 30 May 2025) that integrates four widely used downstream analysis methods into a single automated workflow. scDown is compatible with single-cell datasets analyzed in both Seurat and Scanpy, allowing researchers to seamlessly transition between different single-cell analysis frameworks. This package enables users to perform multiple downstream analyses without requiring extensive programming expertise, making it a valuable resource for the single-cell research community. Additionally, scDown integrates several advanced tools, including scProportionTest [4], which quantifies relative differences in cell proportions for each cluster between two biological conditions; CellChat [5,6], which enables the inference and visualization of complex cell–cell communication networks through ligand–receptor interactions, providing deeper insights into cell interactions across different cell types, tissues, and disease conditions; Monocle3 [7,8,9,10], which performs cell trajectory construction and pseudotime analysis by identifying gene expression changes in a 2D space (UMAP or t-SNE), allowing researchers to study dynamic biological processes such as cell differentiation and development; and scVelo [11,12,13], which enables RNA velocity analysis to predict cellular state transitions and future states and is utilized for visualization, trajectory inference, and probabilistic graph abstraction (PAGA) to explore lineage relationships, cell differentiation, and dynamic transitions in scRNA-seq data. By automating complex multi-step analyses with minimal coding requirements, scDown significantly reduces the time and effort required for experimental biologists, clinicians, and early-career researchers to perform in-depth single-cell RNA-seq analyses. Moreover, its compatibility with both Seurat and Scanpy fosters collaboration across research groups with different software preferences, without compromising reproducibility or analytical rigor.

## 2. Results

### 2.1. Functional Features of scDown

**Figure 1:**
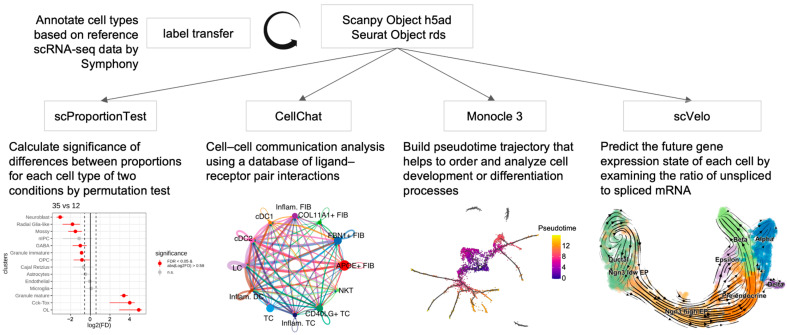
Flowchart of scDown. The scDown pipeline integrates multiple downstream analyses for scRNA-seq data. It includes automated cell type annotation using Symphony, cell proportion comparison with scProportionTest, cell–cell communication analysis via CellChat, trajectory inference with Monocle3, and RNA velocity analysis with scVelo.

scDown accepts scRNA-seq data in both RDS and h5ad format, regardless of cell type annotation status or the integration method used, including Harmony, Canonical Correlation Analysis (CCA), or Batch Balanced K-Nearest Neighbors (BBKNN) [14,15,16]. For unannotated scRNA-seq data, scDown includes a function doTransferLabel to analyze these data by transferring annotations from a reference scRNA-seq dataset, which has similar cell populations using symphony [17]. scDown comprises five modules with each integrating a different published tool (see Figure 1). Module 1: Transfer annotation from a reference scRNA-seq dataset using Symphony; Module 2: Cell proportion differentiation analysis for a given cell type between different conditions using scProportionTest; Module 3: Cell–cell communication analysis for a single or multiple conditions using CellChat (Figure S1); Module 4: Pseudotime analysis using Monocle3 (Figure S2); Module 5: RNA velocity analysis using scVelo (Figure S3). To better guide users in selecting the appropriate modules for their scRNA-seq data, we summarize their utilization in Table 1. Besides integrating different published tools, scDown increases the computational efficiency of running the tools by using parallelized processes which optimize its performance specifically for large datasets (see Table S1). For each key function, the results, including RDS objects, CSV tables, and high-resolution PNG files, are automatically saved in the user-defined directories for reproducibility and publication. Detailed parameters and usage examples for each key function are provided in Figures S1–S3.

### 2.2. Case Study—Application to the Published Dataset

To demonstrate the functions of scDown, we applied it to a published rare disease scRNA-seq dataset (see Section 4). This dataset includes human post-mortem brain samples obtained from the frontal cortex, collected from three biological conditions: CNV (15q duplication syndrome), ASD (autism spectrum disorder without 15q duplication), and CON (neurotypical control) with 17 annotated cell types [18]. We first analyzed cell type proportion differences across three pairwise comparisons (ASD vs. CNV, ASD vs. CON, and CNV vs. CON) to assess variations in cell proportions per each cell type. The ASD group showed a higher proportion of Excitatory Neurons—Layers 5/6 (Neu L56)—cells compared to CON and CNV, with a slightly higher proportion in CON than CNV. Inhibitory Neurons—Vasoactive Intestinal Peptide-positive (Inh-VIP)—cells were more abundant in CNV than in ASD and CON, while Oligodendrocyte Lineage (OL) cells had a lower proportion in CON compared to ASD and CNV (Figure S4). Although the findings of differences in cell proportion should be treated cautiously given potential stochastic variation between individual samples [19], differences in Neu L56 cells are particularly intriguing given the excitatory–inhibitory imbalance theories underlying neurodevelopmental disorders [20,21]. We also identified differences in T-cell proportions in CNV cases vs. either CON control or ASD (Figure S4), which is of interest given the findings below regarding unique neuroinflammation signatures in the CNV condition.

**Figure 2:**
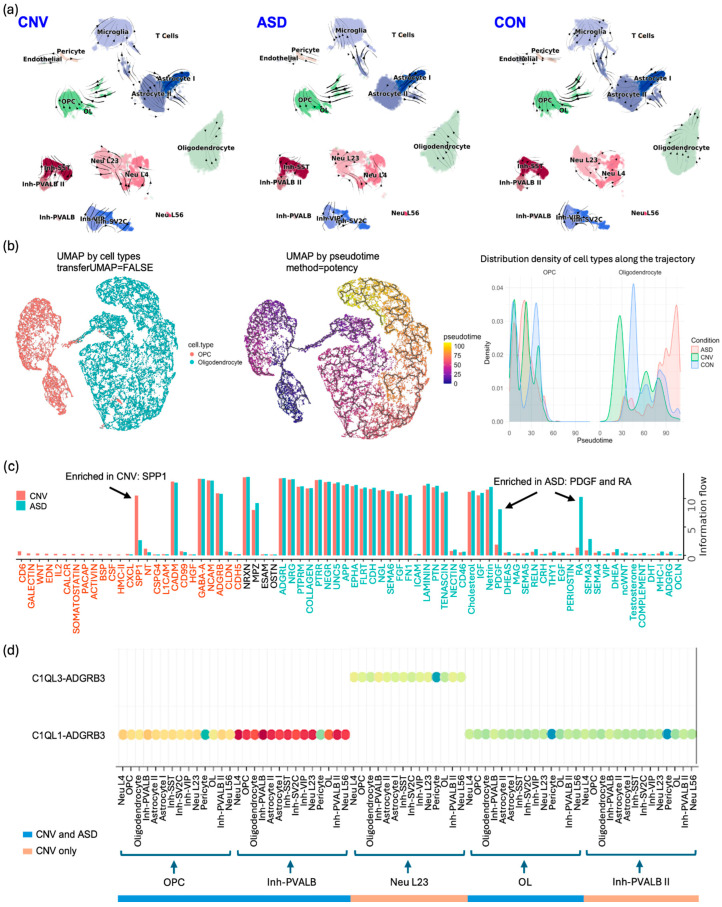
The case study results of single-cell data analysis using the scDown pipeline. (**a**) RNA velocity vector fields are analyzed and visualized on UMAP embeddings separately for each of the three sample conditions (CNV, ASD, and CON). (**b**) The UMAPs show OPCs and oligodendrocytes and their pseudotime trajectories inferred by fitting a principal graph within each partition. Cells were ordered in pseudotime based on a root node identified using maximal potency. The density plot shows the distribution of cells across the pseudotime for the complete dataset separated by the conditions. (**c**) The bar plot compares the relative importance of different signaling pathways between the CNV and CON groups. The *x*-axis represents “Information Flow”, indicating the absolute communication strength of each pathway and highlighting the most influential pathways mediating communication between the groups. The SPP1 pathway is more enriched in CNV samples compared to ASD samples, whereas the PDGF and RA pathways are more enriched in ASD samples than in CNV samples. (**d**) This bubble plot illustrates the ligand–receptor pairs involved in the ADGRB signaling pathway. Each bubble represents a ligand–receptor interaction between a pair of cell types, with its size indicating the significance (*p*-value) of the interaction and its color representing the communication probability.

We performed RNA velocity analysis (Figure 2a) and observed differentiation between Astrocyte I and II subtypes, OL and Oligodendrocyte Progenitor Cells (OPCs), and Inhibitory Neurons—Parvalbumin-positive Type II (Inh-PVALB II)—and Inhibitory Neurons—Somatostatin-positive (Inh-SST). When analyzing CNV, ASD, and CON samples separately, the differentiation between Astrocyte I and II subtypes and OL and OPCs remained consistent across all groups. However, we observed a faster transition from Excitatory Neurons—Layers 2/3 (Neu L23)—to Excitatory Neurons—Layer 4 (Neu L4)—in CNV, as indicated by the longer velocity arrows, followed by ASD, compared to CON samples. This could suggest accelerated neuronal differentiation in CNV which specifically affects Excitatory Neurons. There was also a flow from Inh-PVALB II to Inh-SST in CNV, absent in the other conditions, potentially indicating a disturbance in PVALB and SST interneurons unique to this condition. Importantly, the Astrocyte I-to-Astrocyte II transition was observed in all samples, suggesting protoplasmic and fibrous astrocytes remain distinct in ASD, CNV, and CON. Notably, we also identified an Astrocyte II to Microglia flow, which may indicate shared gene expression profiles between these cell types. Apart from these findings, we identified the top genes with differential RNA velocity for each cell type, providing further insights into the molecular mechanisms driving these patterns (Figure S5).

To explore cell lineage relationships and differentiation progression, we further reconstructed pseudotime trajectories to order cells along developmental paths in which we defined OPCs as the early cell type from which oligodendrocytes differentiate along pseudotime (Figure 2b). In oligodendrocytes, the ASD group exhibited a higher cell density at late stages compared to the other groups. We identified that the top 10 significant trajectory variable genes were highly expressed during early differentiation stages, with higher expression in OPCs compared to oligodendrocytes.

Finally, we investigated and compared the cell–cell communication patterns across different conditions separately. At the aggregated view level, there were no significant differences in the overall strength of incoming and outgoing interactions across all cell types among the three conditions. However, when analyzing specific pathways, we identified condition-enriched pathways in certain cell types. For example, as shown in Figure 2c, the SPP1 (Secreted Phosphoprotein 1) pathway was enriched in CNV, while the PDGF (Platelet-Derived Growth Factor) and RA (Retinoic Acid) pathways were enriched in ASD, corroborating the inflammation signature observed in Dias et al.’s publication and providing new directions for further investigation [18]. Additionally, for pathways enriched in both CNV and ASD, we observed differences in the ligand–receptor pairs involved in cell–cell communication across different cell types. As shown in Figure 2d, the ADGRB (Adhesion G protein-coupled receptor B) pathway exhibited distinct signaling patterns: in CNV, the L23 population communicated with other cell types uniquely through C1QL3/ADGRB3 signaling; in CNV and ASD, C1QL1/ADGRB3 signaling was also present; however, the Inh-PVALB II population uniquely utilized this signaling only in CNV. These results highlight the condition-specific cell–cell communication of synapse regulation, suggesting potential functional implications in disease pathology.

## 3. Discussion

We developed a comprehensive and intuitive scDown tool to streamline scRNA-seq downstream analyses, even for users with limited programming knowledge. To the best of our knowledge, no similar scRNA-seq downstream tools in R or Python have been published yet. While the Shaoxia tool, a web-based application, performs similar analyses, it has several limitations [22]. Firstly, Shaoxia requires users to sign up for an account. Secondly, it does not allow users to directly choose their parameters. For instance, users cannot select their dimensionality parameters for UMAP or t-SNE plots, which are crucial for achieving better cell type annotations. Lastly, Shaoxia requires users to upload their research data to its web server, which may deter researchers with data privacy concerns. In contrast, we created a Docker image for users to install scDown on their laptops, Linux server, or HPC clusters. We also allowed users to customize their parameters according to their specific research objectives.

To assess the differences in cell type proportion between biological conditions, we utilized scProportionTest, a fast R-based tool well integrated with our R-based scDown pipeline. While alternative methods like scCODA, a Bayesian model for compositional single-cell data analysis, are available, they are Python-based and less compatible with our current R framework [23]. For cell–cell communication analysis, we selected CellChat due to its comprehensive pathway-level analysis, support for quantitative comparisons between cell groups, and high-quality visualization. Compared to other tools like NicheNet and SingleCellSignalR, CellChat offers more detailed signaling inference at the pathway level and better integration with Seurat downstream analysis [24,25]. While methods like CellPhoneDB and NATMI are also widely used, they are Python-based and thus less compatible with our R-based pipeline [26,27]. For trajectory and pseudotime analysis, we used Monocle3, which provides extensive downstream analysis capabilities, including the identification of trajectory-associated genes, gene module clustering, and differential expression analysis. The monocle3 framework also offers greater flexibility in root node selection and provides more advanced visualization tools compared to alternative tools such as Slingshot, Palantir, and SCORPIUS [28,29,30]. Finally, for RNA velocity, we adopted scVelo, which improves upon the original velocyto method by extending the model beyond the steady-state assumption through a likelihood-based dynamic modeling approach [31]. scVelo supports multiple modes, including stochastic, dynamical, and deterministic, offering a balance between efficiency and accuracy. scVelo also infers a latent time that enables the coherent temporal ordering of cells based on their transcriptional activity. Compared to tools such as dynamo and DeepVelo, scVelo is more widely adopted and provides a practical balance of performance, interpretability, and seamless integration with our downstream workflows [32,33].

Despite the complex and heterogeneous cell populations in rare diseases, which result in the interplay of different cell types within their cellular environments, scDown was successfully applied to a rare genetic neurodevelopmental disorder in our case study. It detected cell proportion differences, cell lineage relationships, differentiation progression, and cell–cell communication patterns across different conditions separately. scDown can be broadly utilized in downstream analyses of other scRNA-seq data.

To further enhance the utility of our pipeline, we plan to integrate multi-omics data, such as single-cell ATAC-seq, to provide a more comprehensive view of the cellular regulatory mechanisms. We also aim to incorporate additional cell type annotation tools, such as SingleR or Azimuth to complement Symphony and further enhance scalability and robustness [34,35]. Furthermore, adding single-cell variant calling using scRNA-seq data, by leveraging tools such as SComatic and MonoPogen, will allow us to capture genetic variations alongside transcriptional heterogeneity, offering deeper insights into cell type-specific or condition-specific mutations and disease mechanisms [36,37].

## 4. Materials and Methods

### 4.1. Case Study Datasets

We followed the data preprocessing steps outlined in this study to ensure high-quality scRNA-seq analysis [18]. The final integrated dataset consisted of 20 samples, 78,815 cells, and three conditions: CNV, ASD, and CON. Raw sequencing reads were filtered to remove low-quality reads and potential contaminants, ensuring reliable downstream analysis. Dimensionality reduction techniques, such as t-SNE, were applied to reduce data complexity, enabling the visualization of cellular heterogeneity and the identification of distinct cell populations. Identified cell clusters were then annotated based on known marker genes, facilitating the classification of neuronal and glial subtypes. 

### 4.2. Cell Proportion Differentiation Analysis

To assess differences in cell composition between conditions, we utilized the scProportionTest algorithm within our scDown pipeline. The scProportionTest algorithm employs a permutation-based approach to statistically evaluate differences in cell proportions between groups, leveraging Monte Carlo sampling to estimate the significance of the observed differences. In the scDown pipeline, we developed a custom function, run_scproportion, which takes a Seurat object (RDS format) as input along with the names of the metadata columns that define the cell clusters and the sample groups for comparison. The analysis workflow involves creating a scProportion object and performing a permutation test for all pairwise comparisons between sample groups in parallel. This parallelized approach improves computational efficiency, particularly for large datasets. Additionally, users have the flexibility to specify two groups of interest for focused comparisons, allowing for targeted analysis. The function outputs both visualization and statistical results for each comparison. Figures are generated to illustrate the differences in cell proportions between groups, while tables of statistical results provide detailed information on the significance of observed differences.

### 4.3. Cell–Cell Communication Analysis

We developed an automated CellChat analysis framework which streamlines and enhances comprehensive cell–cell communication analysis across different samples or cell groups. This framework allows users to perform comparative analysis, such as investigating differences between disease and control conditions to highlight condition-specific signaling patterns. Notably, we implemented parallel computing to perform the analysis across multiple sample conditions which significantly reduces the computational time and improves the overall efficiency of the workflow. In addition to global analysis, we also allowed users to focus on the particular cell types of interest to investigate how they engage in signaling networks, providing a deeper understanding of their specific roles in cell–cell communication. Additionally, users can also explore the selected pathways of interest to investigate their role in the signaling process. The framework provides various visualizations for these pathways to effectively present and interpret the findings.

### 4.4. Pseudotime Analysis

We developed run_monocle3(), a comprehensive and automated function for pseudotime analysis, using the Monocle3 package v1.3.7 (https://cole-trapnell-lab.github.io/monocle3/, accessed on 30 May 2025), to infer cell differentiation trajectories [7,8]. Our function run_monocle3 takes a Seurat or Scanpy object annotated with cell type labels as the input and applies standard Monocle analysis, including preprocessing, trajectory inference, pseudotime calculation, and the identification of significant genes along trajectory. We provide users the option of (1) the automatic identification of the root node based on the potency from their gene expression profiles and a protein–protein interaction network [9], or (2) selecting the node most heavily occupied by early time point cells, or (3) manually specifying their preferred root node. If biological conditions are specified, the function will also execute Monocle3 separately for each condition in parallel, significantly reducing computation time. Additionally, users can perform pseudotime analysis in parallel for specific combinations of cell types by specifying desired subsets. This function streamlines pseudotime analysis with automated root selection and parallelized condition-specific and subset-specific trajectory inference enhancing scalability and efficiency.

### 4.5. RNA Velocity Analysis

To investigate the dynamics of cellular transitions, we developed two custom functions for RNA velocity analysis using the Python package scVelo v0.3.0 [13]. RNA velocity models splicing kinetics to infer the directionality of cell state transitions.

The first function, run_scvelo(), employs the R package velocyto.R v0.6 [11] and integrates the R package velociraptor v1.8.0 (https://github.com/kevinrue/velociraptor, accessed on 30 May 2025), a wrapper for scVelo. It takes a Seurat or Scanpy object annotated with cell type labels as input. We implemented the custom handling of loom files to seamlessly incorporate spliced and unspliced mRNA count matrices from single or multiple loom files into a unified Seurat object, matching cell barcodes and file names. This function estimates RNA velocity and computes velocity vector fields on UMAP embeddings not only in all data but also in parallel across different time points or groups, improving computational efficiency. The final Seurat object, enriched with spliced/unspliced counts, is also automatically saved in h5ad format for compatibility with the second function run_scvelo_full(). This R-based function streamlines RNA velocity and enhances scalability, enabling the study of transcriptional dynamics at both global data and specific time points.

While the first function, run_scvelo(), relies on an existing R package velociraptor to wrap scVelo, this approach is limited to a subset of scVelo’s capabilities. In contrast, run_scvelo_full() was developed to overcome this limitation by supporting the complete scVelo workflow, including advanced visualizations and inference of trajectories using Partition-based Graph Abstraction (PAGA) [12]. This function automatically loads the h5ad file generated by run_scvelo(), which contains both cell type annotation and spliced/unspliced counts, and performs standard scVelo analysis. In addition, this function performs these analyses for both all data and specific time points or specified conditions. This python-based R function leverages advanced visualizations from scVelo, complementing the streamlined processing in run_scvelo().

## 5. Conclusions

scDown provides a comprehensive and intuitive pipeline for scRNA-seq downstream analysis, integrating cell composition analysis, cell–cell communication analysis, trajectory analysis, and RNA velocity modeling into a single R package. By simplifying these analyses, scDown facilitates biological discovery and enhances the interpretability of single-cell datasets. We applied scDown to a published dataset and identified a unique, previously undiscovered signature of neuronal inflammatory signaling associated with a rare genetic neurodevelopmental disorder. This R package version 1 is freely available at https://github.com/BCH-RC/scDown, accessed on 30 May 2025.

## Figures and Tables

**Table 1:** Required scRNA-seq data type for each module in scDown.

Module	Function	Function Description	Required scRNA-Seq Data		
			Unannotated Data	Annotated Data	
				One Condition	Two or More Conditions
1	doTransferLabel	Automated cell type annotation by transferring cell type annotation from a reference Seurat object to a query unannotated Seurat object	✓		
2	run_scproportion	Statistically assess the significance of differences in cell type proportions for different condition comparisons			✓
3	run_cellchatV2	Perform comprehensive intercellular communications analysis based on ligand–receptor pair interactions across cell types using CellChat.		✓	✓
4	run_monocle3	Construct pseudotime trajectories to model the progression of cellular differentiation utilizing monocle3		✓	✓
5	run_scvelo	Incorporate spliced and unspliced counts using velocyto.R and estimate RNA velocity utilizing velociraptor		✓	✓
	run_scvelo_full	Conduct RNA velocity analysis with enhanced visualizations and PAGA trajectory inference using scVelo		✓	✓

## Data Availability

Raw sequencing data in this case study are available through dbGAP (Access number phs000639v3p1: https://www.ncbi.nlm.nih.gov/gap/advanced_search/?TERM=phs000639v3p1, accessed on 30 May 2025).

## Supplementary Materials

The following supporting information can be downloaded at https://www.mdpi.com/article/10.3390/ijms26115297/s1.
