# Supplementary material for: scDown: A Pipeline for Single-Cell RNA-Seq Downstream Analysis
Source: Int J Mol Sci. 2025 May 30;26(11):5297. doi: 10.3390/ijms26115297 (PMC12154318; doi:10.3390/ijms26115297)
Supplement: Supplementary file 1 [file ijms-26-05297-s001.zip › ijms-3631486-supplementary.pdf]

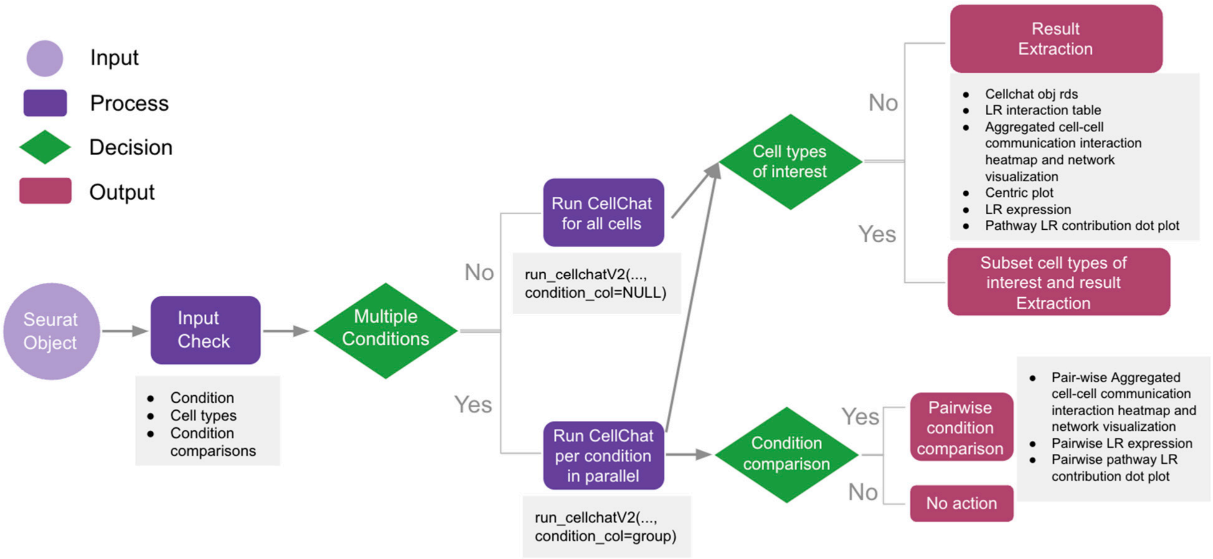

Figure S1: Workflow for Cell-cell Communication Analysis Using CellChat V2. Starting with the Seurat object, we first assess whether sample conditions, cell types or condition comparison parameters are defined. If no sample condition is specified, CellChat analysis is performed on all cells combined. When multiple conditions are present, CellChat is run separately for each condition using parallel computing. If specific cell types are of interest, a subset of cells is extracted for the analysis. Additionally, for datasets with multiple conditions, pairwise comparisons between conditions are performed. All results including rds objects, tables and figures are automatically saved. Detailed parameters and usage examples are provided in the vignette for the scDown::run_cellchatV2 function on Github at https://html-preview.github.io/?url=https://github.com/BCH-RC/scDown/blob/main/vignettes/scDown_CellChatV2.html.

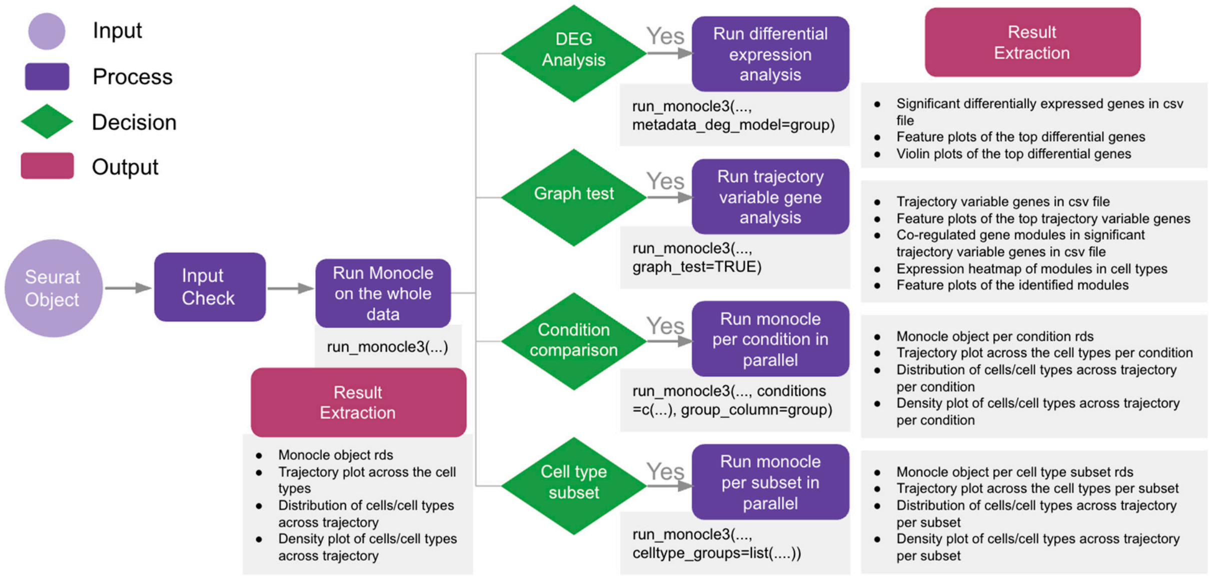

Figure S2: Workflow for Pseudotime Analysis using Monocle3. Starting with a Seurat object, gene expression data is converted to a CDS object for Monocle3, and pseudotime trajectory is performed on the entire dataset. When metadata_deg_model is specified, DEGs between conditions are identified. An option is also provided to analyze trajectory variable genes. When multiple conditions are present, pseudotime analysis is additionally run separately for each condition using parallel computing. If specific cell type subsets are provided, pseudotime analysis is performed for each subset in parallel. All results including rds objects, tables and figures are automatically saved. Detailed parameters and usage examples are provided in the vignette for the scDown::run_monocle3 function on Github at https://html-preview.github.io/?url=https://github.com/BCH-RC/scDown/blob/main/vignettes/scDown_monocle.html.

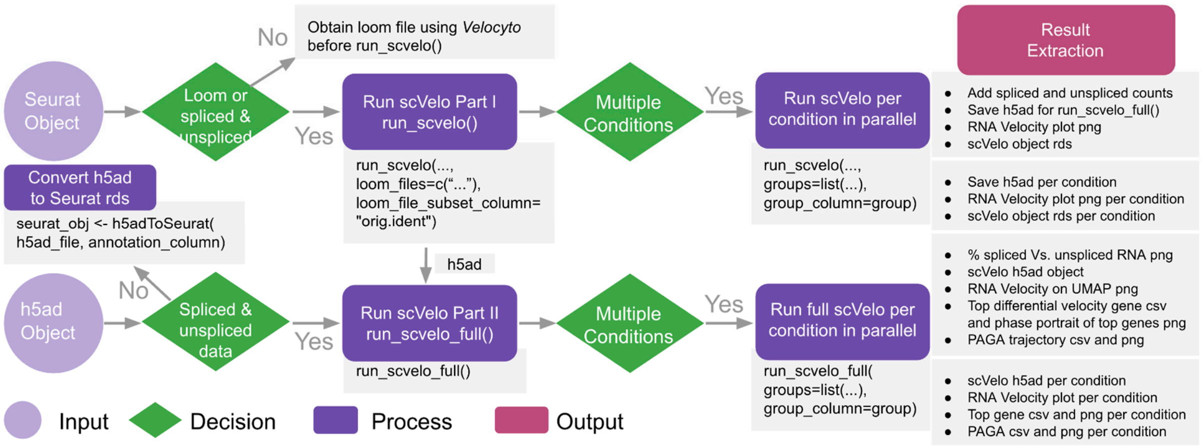

Figure S3: Workflow for RNA Velocity Analysis using scVelo. Starting with a Seurat object and loom files containing spliced and unspliced matrices, the spliced and unspliced counts are first incorporated into the Seurat object as input for run_scvelo(), which is then converted to an h5ad file as input for run_scvelo_full(). RNA velocity analysis is performed on the entire dataset. When multiple conditions or subsets are present, RNA velocity analysis is additionally run separately for each condition or subset using parallel computing. The run_scvelo_full() function further visualizes the percentage of spliced and unspliced RNA per cell type, velocity stream plots, phase portraits of top velocity genes, and PAGA trajectories. All results including rds objects, h5ad objects, tables and figures are automatically saved. Detailed parameters and usage examples are provided in the vignette for the scDown::run_scvelo function on Github at https://html-preview.github.io/?url=https://github.com/BCH-RC/scDown/blob/main/vignettes/run_scvelo.html and the vignette for the scDown::run_scvelo_full function at https://html-preview.github.io/?url=https://github.com/BCH-RC/scDown/blob/main/vignettes/run_scvelo_full.html.

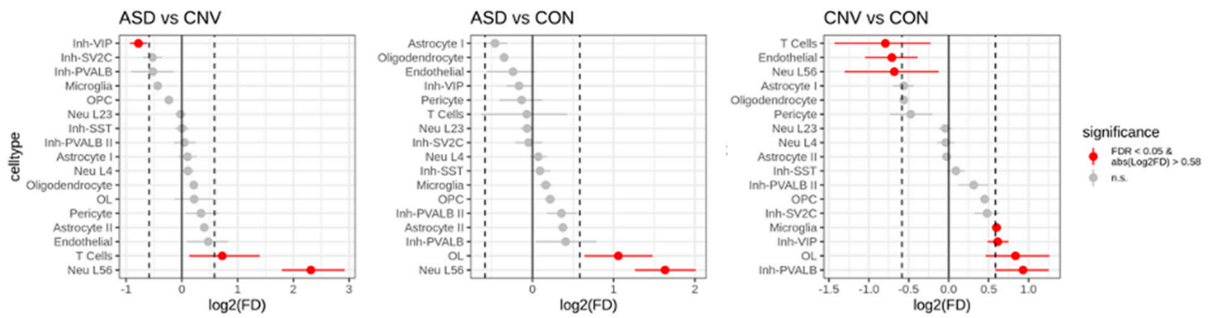

Figure S4: Pairwise Comparisons of Cell Type Proportion Differences in Case Study using scDown. The point-range plot of Cell type proportion differences for pairwise comparisons (ASD vs. CNV, ASD vs. CON, and CNV vs. CON). Neu L56 has the highest cell proportion in ASD samples in comparison to CNV and CON samples.

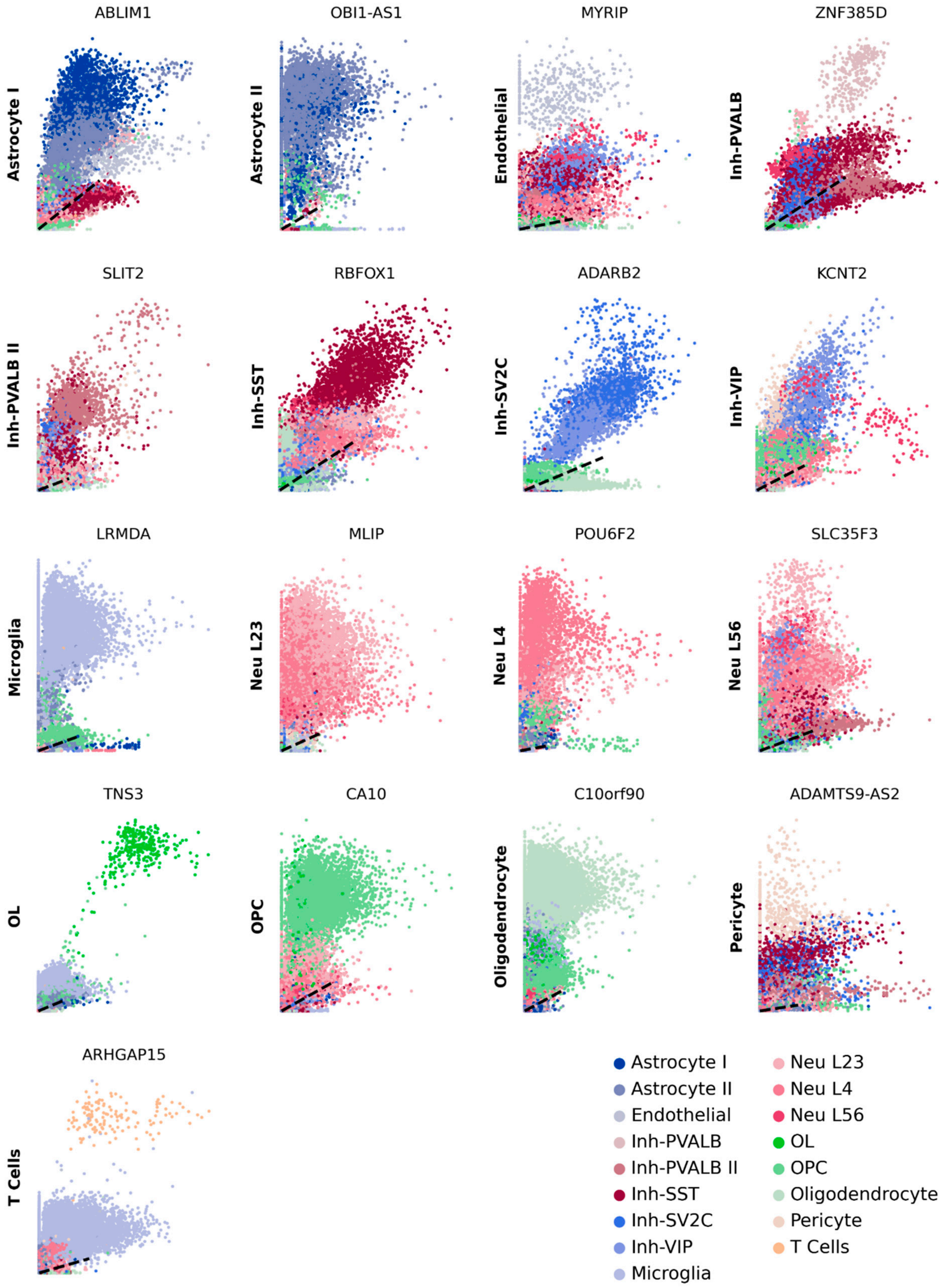
ABLIM1
OBI1-AS1
MYRIP
ZNF385D
Astrocyte I
Astrocyte II
Endothelial
Inh-PVALB
SLIT2
RBFOX1
ADARB2
KCNT2
Inh-PVALB II
Inh-SST
Inh-SV2C
Inh-VIP
LRMDA
MLIP
POU6F2
SLC35F3
Microglia
Neu L23
Neu L4
Neu L56
TNS3
CA10
C10orf90
ADAMTS9-AS2
OL
OPC
Oligodendrocyte
Pericyte
ARHGAP15
T Cells
Astrocyte I
Astrocyte II
Endothelial
Inh-PVALB
Inh-PVALB II
Inh-SST
Inh-SV2C
Inh-VIP
Microglia
Neu L23
Neu L4
Neu L56
OL
OPC
Oligodendrocyte
Pericyte
T Cells

Figure S5: Phase Portraits of Top-likelihood Transcriptionally Induced Genes for Each Cell Type for Case Study using scDown. The phase portrait of unspliced mRNAs (y-axis) and spliced mRNAs (x-axis) is visualized for a top velocity gene for each cell type for the case study data. Transcriptional induction for a particular gene results in an increase of newly-transcribed unspliced mRNAs.

Table S1: Running time before and after parallelization in scDown. Test data (2,930 cells) and case study data (78,815 cells) were analyzed on a High Performance Computing (HPC) Cluster using 32 GB memory with 8 CPUs and 800 GB memory with 8 CPUs respectively. Running time was assessed before and after parallelization.

**scDown modules**	**Running time before parallelization**		**Running time after parallelization**	
	**Test data**	**Case study data**	**Test data**	**Case study data**
Cell proportion difference analysis	22 sec	108 sec	11 sec	88 sec
CellChatV2	12 min	1 hr 10 min	6 min	38 min
Pseudotime analysis	10 min	10 hr 43 min	5 min	3 hr 52 min
RNA velocity analysis	2 min	2 hr	1 min	1 hr 20 min
